# An Efficient Augmented Lagrangian Method for Statistical X-Ray CT Image Reconstruction

**DOI:** 10.1371/journal.pone.0140579

**Published:** 2015-10-23

**Authors:** Jiaojiao Li, Shanzhou Niu, Jing Huang, Zhaoying Bian, Qianjin Feng, Gaohang Yu, Zhengrong Liang, Wufan Chen, Jianhua Ma

**Affiliations:** 1 School of Biomedical Engineering, Southern Medical University, Guangzhou 510515, China; 2 School of Mathematics and Computer Sciences, Gannan Normal University, Ganzhou 341000, China; 3 Department of Radiology, State University of New York, Stony Brook, NY 11794, United States of America; Chongqing University, CHINA

## Abstract

Statistical iterative reconstruction (SIR) for X-ray computed tomography (CT) under the penalized weighted least-squares criteria can yield significant gains over conventional analytical reconstruction from the noisy measurement. However, due to the nonlinear expression of the objective function, most exiting algorithms related to the SIR unavoidably suffer from heavy computation load and slow convergence rate, especially when an edge-preserving or sparsity-based penalty or regularization is incorporated. In this work, to address abovementioned issues of the general algorithms related to the SIR, we propose an adaptive nonmonotone alternating direction algorithm in the framework of augmented Lagrangian multiplier method, which is termed as “ALM-ANAD”. The algorithm effectively combines an alternating direction technique with an adaptive nonmonotone line search to minimize the augmented Lagrangian function at each iteration. To evaluate the present ALM-ANAD algorithm, both qualitative and quantitative studies were conducted by using digital and physical phantoms. Experimental results show that the present ALM-ANAD algorithm can achieve noticeable gains over the classical nonlinear conjugate gradient algorithm and state-of-the-art split Bregman algorithm in terms of noise reduction, contrast-to-noise ratio, convergence rate, and universal quality index metrics.

## Introduction

Statistical iterative reconstruction (SIR) approaches for X-ray computed tomography (CT) using the penalized weighted least squares (PWLS) criteria [1–6], which model the statistical properties of the measurements and impose adequate penalty or regularization on objective function, have shown their sophisticated ability in achieving a superior noise-resolution tradeoff as compared with analytical reconstruction approaches such as the well-known filtered back-projection (FBP) algorithm. Generally, the SIR methods can be derived from the maximum a posteriori (MAP) estimator as given the observed data or measurement, and consist two terms (i.e., data fidelity and penalty terms) in the associative objective function. Practically, due to the nonlinear expression of the objective function, PWLS-based SIR methods often suffer from the heavy computational load and slow convergence rate [2, 7].

Up to now, for yielding a high-quality CT image, several types of the statistical iterative reconstruction algorithms have been proposed. For example, Sauer and Bouman [8] proposed a Gauss-Seidel (GS) algorithm with the flexible ability to incorporate some general prior models, including Gaussian Markov and non-Gaussian priors, which was successfully used in low-dose cone-beam CT image reconstruction [2]. Thibault [9] described a coordinate descent algorithm with significant gains over direct analytical methods in terms of noise reduction, resolution preservation, and artifacts suppression. To achieve fast convergence rate, Benson *et al* [10] presented a block-based coordinate descent algorithm and Fessler and Booth [7] proposed a conjugate gradient preconditioning algorithm. Recently, by facilitating the objective function optimization in image processing and medical imaging, the variable splitting approaches have been widely studied with remarkable advantages [4, 11–18]. Specifically, the variable splitting approaches render the resulting constrained problem tractable to an alternating minimization scheme, which can simplify and decouple the optimization with respect to auxiliary variables and simplify optimization. A typical example is that, Ramani and Fessler [4] proposed an accelerated alternating direction method of multipliers for CT image reconstruction via an improved variable splitting scheme to optimize the PWLS cost function. The associative experiments demonstrated the variable splitting scheme can achieve remarkable gains over other general algorithm in CT image reconstruction in term of convergence rate and computation time.

Inspired by the nature of the variable splitting scheme [12], in this work, we propose an adaptive nonmonotone alternating direction optimization strategy via an efficient augmented Lagrangian multiplier method, which is termed as “ALM-ANAD”. We apply it to minimizing the PWLS cost function, aiming to address issues of the algorithms related to the SIR for CT image reconstruction. The alternating direction technique [19] and adaptive nonmonotone line search scheme [20, 21] are adapted into the ALM-ANAD algorithm with accelerated convergence rate for CT image reconstruction. Qualitative and quantitative evaluations are carried out on both the digital and patient data in terms of different image quality measure criteria.

The remaining part of the paper is organized as follows. Section 2 describes the present ALM-ANAD algorithm. In Section 3, the ALM-ANAD algorithm is applied to solving the PWLS minimization problem in statistical X-ray CT image reconstruction. The experiments’ setup and evaluation metrics are also presented in this section. In Section 4, the evaluation of the present algorithm is performed on both digital and physical phantoms, followed by the discussion and conclusion in Section 5.

## Proposed algorithm

### Preliminary results

Without loss of generality, we consider the following equality-constrained non-smooth minimization problem

$$\begin{array}{c} \min_{x,y}f\left(x,y\right),\;\;s.t.\;\;h\left(x,y\right)=0 \end{array}$$ (1)

where the vector-valued function *h* is differentiable with respect to both *x* and *y*, but the function *f* may or may not be differentiable with respect to *y*.

For solving the problem Eq (1), we will propose an algorithm in the framework of the augmented Lagrangian multiplier (ALM) method, first suggested by Hestenes [22] and Powell [23]. In the ALM frame, one obtains the *k*-th iteration *x*_*k*_, *y*_*k*_ by minimizing the following augmented Lagrangian function

$$\begin{array}{c} L\left(x,y,\lambda \right)=f\left(x,y\right)-\lambda^{T}h\left(x,y\right)+\frac{\beta }{2}h{\left(x,y\right)}^{T}h\left(x,y\right),\;\mu >0. \end{array}$$ (2)

jointly with respect to both *x* and *y* for a given multiplier λ = λ_*k* − 1_, the updates the multiplier by λ_*k*_ = λ_*k* − 1_ − *βh*(*x*_*k*_, *y*_*k*_). In the implementation, the complexity of an ALM algorithm depends almost entirely on how the augmented Lagrangian function is minimized jointly with respect to both *x* and *y*. Therefore, we concentrate on solving unconstrained optimization problem as follows:

$$\begin{array}{c} \min_{x,y}\phi \left(x,y\right) \end{array}$$ (3)

where *ϕ* is differentiable with respect to the block variable *x* but not necessarily to *y*. Furthermore, assuming that

$$\begin{array}{c} y\left(x\right)=\arg\min_{y}\phi \left(x,y\right) \end{array}$$ (4)

exists and is unique for each *x* in a region of interest, then the problem of Eq (3) can be rewritten as an unconstrained minimization one with respect to *x*, i.e.,

$$\begin{array}{c} \min_{x}\psi \left(x\right)≜\phi \left(x,y\left(x\right)\right) \end{array}$$ (5)

where *ψ*(*x*) is generally nonsmooth.

### ANAD algorithm

We now present an adaptive nonmonotone line search algorithm for solving the nonsmooth problem Eq (5), which is an extension to the one in [20] designed for minimizing smooth function. The reader interested in this line search strategy can find more information in S1 Text.

Since the cost function *ψ*(*x*) = *ϕ*(*x*, *y*(*x*)) is non-differentiable, the nonmonotone line search algorithm developed by Dai and Fletcher [20] cannot be used directly. To address this issue, we replace the gradient of *ψ*(*x*_*k*_) by ∇_1_ *ϕ*(*x*_*k*_, *y*(*x*_*k*_)), which can be regarded as a subgradient direction for *ψ*(*x*). In the implementation, the search direction was set as *d*_*k*_ = −*t*_*k*_∇_1_ *ϕ*(*x*_*k*_, *y*(*x*_*k*_)), and the new BB step-size *t*_*k*_ is adaptively determined as

$$\begin{array}{c} t_{k}=\{\begin{array}{c} t_{k}^{BB1}\text{,}\;\;\text{if}\;\;\frac{s_{k-1}^{T}y_{k-1}}{{∥s_{k-1}∥}_{2}{∥y_{k-1}∥}_{2}}>\tau ,\\ \min\{t_{j}^{BB2}|j=\max\left\{1,k-h\right\},...,k\}\text{,}\;\text{otherwise,} \end{array} \end{array}$$ (6)

where *τ* ∈ (0, 1) and *h* > 0 is an integer. According to [24], the scalar $t_{k}^{BB1}$ and $t_{k}^{BB2}$ are determined by $t_{k}^{BB1}=s_{k-1}^{T}s_{k-1}/s_{k-1}^{T}y_{k-1}$ and $t_{k}^{BB2}=s_{k-1}^{T}y_{k-1}/y_{k-1}^{T}y_{k-1}$ where *s*_*k* − 1_ = *x*_*k*_ − *x*_*k* − 1_, *y*_*k* − 1_ = ∇_1_ *ϕ*(*x*_*k*_, *y*_*k*_) − ∇_1_ *ϕ*(*x*_*k* − 1_, *y*_*k*_).

To suite our situation we need to modify the nonmonotone line search into the following form

$$\begin{array}{c} \phi \left(x_{k}+\alpha d_{k},y_{k}\right)\leq \phi_{r}+\alpha \delta \nabla_{1}\phi {\left(x_{k},y_{k}\right)}^{T}d_{k} \end{array}$$ (7)

where *y*_*k*_ = *y*(*x*_*k*_) and *ϕ*_*r*_ is a reference function value.

In summary, our proposed adaptive nonmonotone alternating direction (ANAD) algorithm can be described as follows:

**Step 1: Given** 0 < *δ*, *ρ* < 1, 0 < *ρ*_min_ ≤ *ρ*_max_, 0 < *θ*_1_ ≤ *θ*_2_ < 1,

*ρ* ∈ [*θ*_1_, *θ*_2_], integer *l*, *h*, *K*, *tol* > 0, with *ϕ*_*r*_ = +∞,

*ϕ*_best_= *ϕ*_c_ = (*x*_0_,*y*_0_). Set k = 0;

**Step 2: While** ∥∇_1_ *ϕ*(*x_k_, y_k_*)∥ ≤ *tol* is not met;

**Step 3**: Impose *t*_*k*_ such that *t*_*k*_ ∈ [*ρ*_min_, *ρ*_max_]. Set *d*_*k*_ = −*t*_*k*_∇_1_ *ϕ*(*x*_*k*_, *y*_*k*_) and *α* = 1;

**Step 4**: Compute the step-size *α*_*k*_ with *α*_*k*_ = max{*αρ* ^*i*^:*i* = 0, 1, ⋯} such that

*ϕ*(*x*_*k*_+*αρ* ^*i*^ *d*_*k*_, *y*_*k*_) ≤ *ϕ*_*r*_+*αδρ* ^*i*^∇_1_ *ϕ*(*x*_*k*_, *y*_*k*_)^*T*^ *d*_*k*_;

**Step 5**: Set *x*_*k*+1_ = *x*_*k*_+*α*_*k*_ *d*_*k*_;

**Step 6**: Update the reference function *ϕ*_*r*_:

    If *ϕ*(*x*_*k* + 1_, *y*_*k*_) ≤ *ϕ*_*best*_, then *ϕ*_*best*_ = *ϕ*(*x*_*k*+1_, *y*_*k*_), *ϕ*_*c*_ = *ϕ*(*x*_*k*+1_, *y*_*k*_), *l* = 0;

    Else *ϕ*_*c*_ = max{*ϕ*_*c*_, *ϕ*(*x*_*k*+1_, *y*_*k*_)}, *l* = *l*+1;

    If *l* = *K*, then *ϕ*_*r*_ = *ϕ*_*c*_, *ϕ*_*c*_ = *ϕ*(*x*_*k*+1_, *y*_*k*_), *l* = 0;

    End If

**Step 7**: Compute *y*_*k* + 1_ = *y*(*x*_*k* + 1_) ≜ arg min_y_ *ϕ*(*x*_*k* + 1_, *y*);

**Step 8**: Compute *t_k_* with Eq (6);

**Step 9: End** if stop criterion is satisfy.

The ANAD algorithm differs from the traditional alternating minimization scheme since it does not require exact (or high precision) minimum in all directions, and it differs from the block coordinate descent approach [9], since it does not require a monotone decease of objective function.

### ALM-ANAD algorithm

After embedding the ANAD algorithm into the ALM framework, we can obtain the ALM-ANAD algorithm for solving the equality-constrained minimization problem Eq (1).

**Step 1: Initialize** *β*, *γ* > 0, *x*_0_, *y*_0_ = *Rx*_0_, λ_0_ = 0. Set *k* = 0;

**Step 2: While** stop criterion is not met;

**Step 3**:  Call ANAD algorithm to minimize $\phi (x,y)\;≜\;L(x,y,\lambda_{k})$ starting from (*x*_*k*_, *y*_*k*_),

     giving the output (*x*_*k*+1_, *y*_*k*+1_);

**Step 4**:  Update the multiplier: λ_*k*+1_ = λ_*k*_ − *γ*(*Rx*_*k*+1_ − *y*_*k*+1_);

**Step 5: End** if stop criterion is satisfy.

The selection of *β* and *γ* in Step 1 will be discussed in next section. The iterative process in Step 2 is terminated if certain convergence criteria is satisfied for a relatively stable solution [1, 6]. The convergence of ALM-ANAD algorithm will be analyzed in S2 Text.

## PWLS CT Image Reconstruction with ALM-ANAD Algorithm

### PWLS criteria for statistical X-ray CT reconstruction

The statistical model for X-ary CT projection data after logarithm transform usually follows a Gaussian approximation with a data-dependent variance [1, 5, 6, 25], and the associative variance can be determined by the following analytical formula in our previous work [25]

$$\begin{array}{c} \sigma_{i}^{2}=\frac{1}{I_{0}}\exp\left({\bar{p}}_{i}\right)(1+\frac{1}{I_{0}}\exp({\bar{p}}_{i})(\sigma_{e}^{2}-1.25)) \end{array}$$ (8)

where *I*_0_ is the incident X-ary intensity, ${\bar{p}}_{i}$ is the mean of the sinogram data at bin *i* and $\sigma_{e}^{2}$ is the variance of background electronic noise. As described in detail previously [3], the penalized weighted least-squares (PWLS) cost function for CT image reconstruction with a penalty term ψ (*Rx*) can be expressed as follows:

$$\begin{array}{c} x^{*}=\arg\min_{x}\Psi \left(Rx\right)+\frac{\beta }{2}{\left(p-Hx\right)}^{T}W^{-1}\left(p-Hx\right) \end{array}$$ (9)

where *p* = (*p*_1_, *p*_2_,…, *p*_*M*_)^*T*^ denotes the line integral data (or sinogram data) after system calibration and logarithm transformation, *x* = (*x*_1_, *x*_2_,…, *x*_*N*_)^*T*^ is the vector of attenuation coefficients to be estimated, where “*T*” denotes the matrix transpose. As described in detail previously [3], operator *H* represents the system or projection matrix with the size of *M* × *N*. As described in detail previously [3], the element of *h*_*ij*_ is the length of intersection of projection ray *i* with pixel *j*. In our implementation, as described in detail previously [3], the element of matrix *H* was precalculated with a fast ray-tracing technique [26] and stored as a file. *W* is a diagonal matrix with the *i*th element of $\sigma_{i}^{2}$, which can be estimated from the measured projection data according to Eq (8). *β* is a hyper-parameter to balance the penalty term (the first term of Eq (9)) and the data fidelity term (the second term of Eq (9)).

As for ψ (*Rx*), we consider a general family of penalty with the following form

$$\begin{array}{c} \Psi \left(Rx\right)=\sum_{r=1}^{N_{1}}\Phi_{r}(\sum_{p=1}^{P}|{\left[R_{p}x\right]}^{r}{|}^{m_{1}}) \end{array}$$ (10)

where Φ _*r*_ denotes potential function, [*x*]^*r*^ represents the *r*-th element of vector *x*, and the *N*_2_ *P* × *N* matrix $R={\left[R_{1}^{T},R_{2}^{T},\ldots ,R_{P}^{T}\right]}^{T}$ constitutes shift-invariant operators *R*_*p*_, *p* = 1,⋯, *P* ∀*p* (e.g., tight frames, finite differences) with the size of *N*_2_ × *N*. ψ in Eq (10) is specified as a function of the to-be-reconstructed image *x*, which includes several popular smooth/non-smooth forms as described in [4, 14, 21, 27–29]. For brevity, we only concentrate on following two particular instances of Eq (10) in this study,

- **Smooth edge-preserving regularization**: *m*_1_ = 1, *P* = 2, *R*_1_ and *R*_2_ represent horizontal and vertical finite-differencing matrices, respectively, and Φ _*r*_(*μ*) = *μ*/*s* − log(1 + *μ*/*s*), *s* > 0, where *r* indexes the rows of *R*_1_ or *R*_2_.
- ***ℓ*_1_-regularization**: *m*_1_ = 1, *P* = 2, *R*_1_ and *R*_2_ represent horizontal and vertical finite-differencing matrices, respectively, and Φ _*r*_(*μ*) = *μ*, where *r* indexes the rows of *R*_1_ or *R*_2_.

These regularizers have been successfully applied to PWLS problem in X-ary CT image reconstruction [7, 30]. We note that the difficulties arise when one uses *ℓ*_1_-regularization. This regularizer is not differentiable everywhere precluding optimization by conventional gradient descent methods. Differentiable approximations can be employed, but such modifications will lead to slow convergence of conventional gradient descent methods [4].

### Implementation details

To separate the non-differentiable penalty term in PWLS cost function, we split variable by introducing *y* = *Rx*. Then the problem of Eq (9) can be transformed to an equivalent constrained one with auxiliary constraint variable *y*

$$\begin{array}{c} \min_{x}\Psi \left(y\right)+\frac{\beta }{2}{\left(p-Hx\right)}^{T}W^{-1}\left(p-Hx\right),\;\;s.t.\;y=Rx. \end{array}$$ (11)

The problem of Eq (11) can be regarded as a special form of Eq (1), while the non-differentiable part of the augmented Lagrangian function is easy to solve due to separability.

In the case of solving the problem of Eq (11) with the ALM-ANAD algorithm, we have

$$\begin{array}{ll} \phi \left(x,y\right)≜L\left(x,y,\lambda \right) & =\Psi \left(y\right)-\lambda^{T}\left(Rx-y\right)+\frac{\gamma }{2}∥Rx-y{∥}^{2}\\ & \;+\frac{\beta }{2}{\left(p-Hx\right)}^{T}W^{-1}\left(p-Hx\right). \end{array}$$ (12)

Then, we can easily derive

$$\begin{array}{c} \nabla_{1}\phi \left(x,y\right)=\beta H^{T}W^{-1}\left(Hx-p\right)-R^{T}\lambda +\gamma R^{T}\left(Rx-y\right). \end{array}$$ (13)

Additionally, the minimization of *ϕ*(*x*, *y*) with *y* can be written as follows

$$\begin{array}{c} y\left(x\right)=\arg\min_{y}\phi \left(x,y\right)=\arg\min_{y}\Psi \left(y\right)-\lambda^{T}\left(Rx-y\right)+\frac{\gamma }{2}{∥Rx-y∥}^{2}. \end{array}$$ (14)

For the above two regularizers, Eq (14) can be separated into *P* × *N*_2_ **1D** minimization problems with regard to the component {*y* ^*i*^}

$$\begin{array}{c} \arg\min_{y^{i}}\phi \left(x,y^{i}\right)=\arg\min_{y^{i}}\Psi \left(y^{i}\right)-\lambda_{k}^{i}({\left[Rx\right]}^{i}-y^{i})+\frac{\gamma }{2}{\left({\left[Rx\right]}^{i}-y^{i}\right)}^{2}. \end{array}$$ (15)

For the smooth edge-preserving regularization, the solution of Eq (15) is given with the shrinkage rule [31]

$$\begin{array}{c} y_{k+1}^{i}=sign\left({\left[Rx_{k}\right]}^{i}-\lambda_{k}^{i}/\gamma \right)\frac{z^{i}+\sqrt{{\left(z^{i}\right)}^{2}+4s|{\left[Rx_{k}\right]}^{i}-\lambda_{k}^{i}/\gamma |}}{2} \end{array}$$ (16)

where $z^{i}=|{\left[Rx_{k}\right]}^{i}-\lambda_{k}^{i}/\gamma |-s-1/s\gamma$. Similarly, for the analysis *ℓ*_1_-regularization, the solution of Eq (15) is

$$\begin{array}{c} y_{k+1}^{i}=\max\{|{\left[Rx_{k}\right]}^{i}-\lambda_{k}^{i}/\gamma |-1/\gamma ,0\}sign({\left[Rx_{k}\right]}^{i}-\lambda_{k}^{i}/\gamma ). \end{array}$$ (17)

### Selection of *β* and *γ*

In general, choosing appropriate values for penalty parameter is a nontrivial and application-dependent task. For the ALM-ANAD algorithm, the penalty parameter *β* in Eq (9) controls the relative contributions of the two terms, i.e., the data-fidelity term and penalty term. Because the data fidelity term is proportional to the noise standard deviation in the projection domain, *β* should be increased with the noise increment. In practice, the penalty parameter *β* can be determined through an empirical, subjective, and time consuming trial and error process [32]. In this study, extensive experiments illustrated that the value of *β* within the range from 100 to 10000 was proper for both NCG, SB-NCG and ALM-ANAD algorithms.

For penalty parameter *γ*, we use an empirical rule that is based on [12]. It was suggested in [12] that we can therefore choose a value for *γ* that minimizes the condition number of the subproblem Eq (5), resulting in fast convergence for iterative optimization methods. According to distance-driven (DD) projector [33] and *R* in Eq (14), the minimum condition number *υ*_min_ ≈ 10^5^, which subsequently resulted in slow convergence of ALM-ANAD in our experiments. Based on our experience with CT image reconstruction experiments, we found the empirical rule *γ* = *υ*_min_/100 can yield good overall convergence speeds for ALM-ANAD algorithm.

### Comparison methods and other experiments setting

To validate and evaluate the performance of the present ALM-ANAD algorithm for X-ray CT image reconstruction, the nonlinear conjugate gradient (NCG) [34] and split-Bregman algorithms [12] are adopted for comparison.

The NCG algorithm is an efficient approach that monotonically decrease the PWLS cost function Eq (9). The search direct *d*_*k*_ is generated by the following way

$$d_{k}=\{\begin{array}{ll} -g_{k}, & k=0\\ -g_{k}+\beta_{k}d_{k-1}, & k\geq 1 \end{array}$$ (18)

where *g*_*k*_ is the gradient of the PWLS cost function at *x*_*k*_, $\beta_{k}=\frac{g_{k}^{T}\left(g_{k}-g_{k-1}\right)}{g_{k-1}^{T}g_{k-1}}$.

The split-Bregman iteration for problem Eq (11) is stated as follows:

$$\begin{array}{ccc} x_{k+1} & = & \arg\min_{x}\frac{\beta }{2}{\left(p-Hx\right)}^{T}W^{-1}\left(p-Hx\right)+\frac{\gamma }{2}{∥y-Rx-b_{k}∥}^{2}, \end{array}$$ (19)

$$\begin{array}{ccc} y_{k+1} & = & \arg\min_{y}\Psi \left(y\right)+\frac{\gamma }{2}{∥y-Rx_{k+1}-b_{k}∥}^{2}, \end{array}$$ (20)

$$\begin{array}{ccc} b_{k+1} & = & b_{k}+\left(Rx_{k+1}-y_{k+1}\right). \end{array}$$ (21)

Due to the subproblem Eq (19) is calculated by the NCG algorithm, the split-Bregman algorithm was termed as “SB-NCG” in this paper. The subproblem Eq (20) can also be solved by the shrinkage rule [31] for the above two regularizers.

The related parameters of above three algorithms in the implementation were selected as follows: for the ALM-ANAD algorithm, (1) the multiplier λ_0_ was initialized as zero; (2) the initial guess *x*_0_ is the result from the FBP method with ramp filter; (3) *ρ*_min_ = 1/*ρ*_max_ = 1.0 × 10^−10^, *δ* = 10^−4^, *tol* = 1.0 × 10^−3^, *ρ* = 0.5, *K* = 5, *h* = 3, for all the cases; (4) The *s*, *β* and *γ* were selected empirically for different cases based on quantitative measures and visual inspection. For the NCG and SB-NCG algorithms, *s* and *β* were also selected empirically for different cases based on quantitative measures and visual inspection. This scheme can also be considered as a trial and error process. For XCAT phantom, we used the smooth edge-preserving regularization, while for clinical data we used *ℓ*_1_-regularization. Since NCG cannot directly handle non-smooth regularization without smoothing it, so we used a smoothing value of 10^−6^ in *ℓ*_1_-regularization.

### Experimental data acquisitions

To validate and evaluate the performance of the present ALM-ANAD algorithm in low-dose x-ray CT image reconstruction, a digital XCAT phantom [35] and an anthropomorphic torso phantom (Radiology Support Devices, Inc., Long Beach, CA) were used for the experiments.

#### Digital XCAT phantom

A slice of the XCAT phantom (Fig 1(a) in [28]) was used, which contains head anatomy structures with a tumor lesion. For the CT projection simulation, we chose a geometry that was representative of a monoenergetic fan-beam CT scanner setup. The imaging parameters of the CT scanner were described in detail previously [3]: (1) each rotation included 1160 projection views that were evenly spaced on a circular orbit; (2) the number of channels per view was 672; (3) the distance from the detector arrays to the X-ray source was 1040 mm; (4) the distance from the rotation center to the X-ray source was 570 mm; and (5) the space of each detector bin was 1.407 mm. All the reconstructed images were composed of 512 × 512 square pixels. The size of each pixel was 0.625 mm × 0.625 mm. Each projection datum along an X-ray through the sectional image was calculated based on the known densities and intersection areas of the ray with the geometric shapes of the objects in the sectional image.

**Fig 1:**
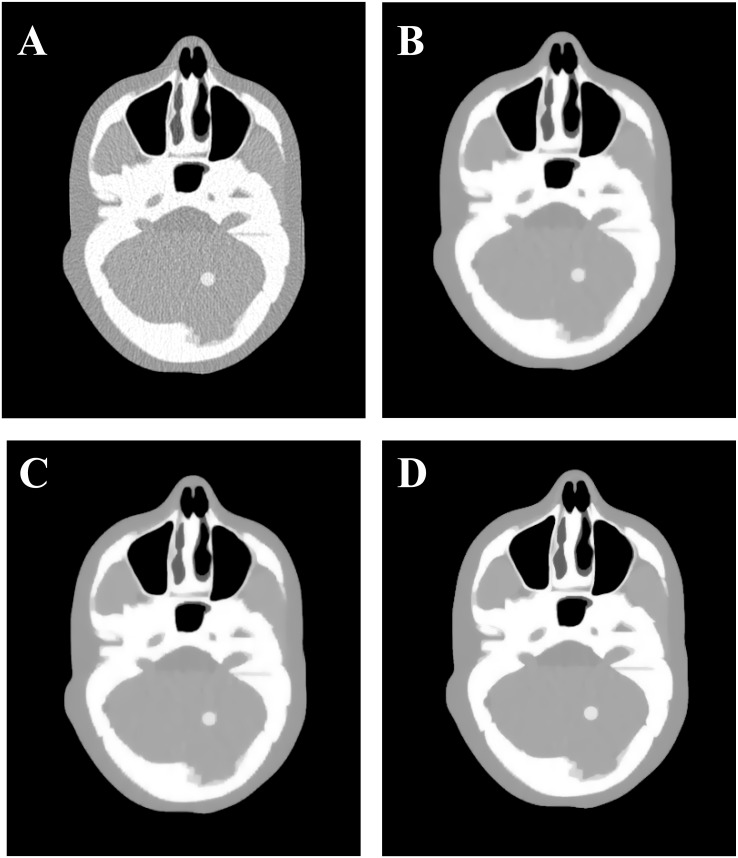
The XCAT phantom results reconstructed by four different algorithms from the same noisy sinogram data. (A) is the result from the FBP method with ramp filter; (B) is the result from the NCG algorithm with *s* = 1.0 × 10^−2^, *β* = 2.0 × 10^4^; (C) is the result from the SB-NCG algorithm with *s* = 1.0 × 10^−3^, *γ* = 128, *β* = 8.0 × 10^3^; and (D) is the result from ALM-ANAD algorithm with *s* = 1.0 × 10^−3^, *γ* = 200, *β* = 2.0 × 10^3^. All images are displayed with the same window [0.0048 0.0128] mm^−1^.

As described in detail previously [3], we first simulated the noise-free sinogram data $\hat{y}$ then generated the noisy transmission measurement *I* according to the statistical model of the pre-logarithm projection data, that is,

$$\begin{array}{c} I=\text{Poisson}(I_{0}\exp(-\hat{y}))+\text{Normal}(0,\sigma_{e}^{2}) \end{array}$$ (22)

where *I*_0_ is the incident X-ray intensity and $\sigma_{e}^{2}$ is the background electronic noise variance. In the simulation, *I*_0_ and $\sigma_{e}^{2}$ were set to 1.0 × 10^5^ and 11.0, respectively. Finally, the noisy sinogram data *y* were calculated by performing the logarithm transformation on the transmission data *I*.

#### Anthropomorphic torso phantom

The anthropomorphic torso phantom (Fig 1(b) in [28]) was used for the experimental data acquisition. The phantom was scanned by a clinical CT scanner (Siemens SOMATOM Sensation 16 CT) at 40 mAs, 120 kVp. The associated imaging parameters of the CT scanner were described in detail previously [3]: (1) each rotation included 1160 projection views that were evenly spaced on a circular orbit; (2) the number of channels per view was 672; (3) the distance from the detector arrays to the X-ray source was 1040 mm; (4) the distance from the rotation center to the X-ray source was 570 mm; and (5) the space of each detector bin was 1.407 mm. All the reconstructed images were composed of 512 × 512 square pixels. The size of each pixel was 1.2 mm × 1.2 mm.

### Performance evaluation

#### Evaluation by noise reduction

The following metrics were utilized to evaluate the noise reduction for the quantitative comparison: (1) signal-to-noise ratio (SNR); (2) mean square error (MSE):

$$\begin{array}{c} \text{SNR}=10\log_{10}\frac{\sum_{m=1}^{N}{\left(x_{\text{true}}\left(m\right)\right)}^{2}}{\sum_{m=1}^{N}{(x_{\text{true}}\left(m\right)-x\left(m\right))}^{2}} \end{array}$$ (23)

$$\begin{array}{c} \text{MSE}=\frac{1}{N}\sum_{m=1}^{N}{\left(x_{\text{true}}\left(m)-x(m\right)\right)}^{2} \end{array}$$ (24)

where *x*(*m*) denotes the voxel value of the reconstructed image at voxel *m*, *x*_true_(*m*) denotes the voxel value of the true phantom image at voxel *m*, and *N* is the total number of voxels in the image.

#### Evaluation by contrast-to-noise ratio

The CNR is defined as follows:

$$\begin{array}{c} \text{CNR}=\frac{\left|x_{\text{ROI}}-x_{\text{BG}}\right|}{\sqrt{\sigma_{\text{ROI}}^{2}+\sigma_{\text{BG}}^{2}}} \end{array}$$ (25)

where *x*_ROI_ denotes the mean of the voxels inside the ROI, and *x*_BG_ denotes the mean of the voxels in the background region. The terms $\sigma_{\text{ROI}}^{2}$ and $\sigma_{\text{BG}}^{2}$ represent the standard deviations of the voxels inside the ROI and the background region, respectively.

#### Image-similarity

To assess the image-similarity between the reconstructed and true images, the universal quality index (UQI) [36] was used in this study. After selected the aligned ROI within the reconstructed and true images, the associative means, variances and covariances over the ROI can be calculated as

$$\begin{array}{c} \bar{x}=\frac{\sum_{m=1}^{Q}x(m)}{Q},\;{\bar{x}}_{\text{true}}=\frac{\sum_{m=1}^{Q}x_{\text{true}}\left(m\right)}{Q} \end{array}$$ (26)

$$\begin{array}{c} \sigma^{2}=\frac{\sum_{m=1}^{Q}{(x\left(m\right)-\bar{x}\left(m\right))}^{2}}{Q-1},\;\sigma_{\text{true}}^{2}=\frac{\sum_{m=1}^{Q}{(x_{\text{true}}\left(m\right)-{\bar{x}}_{\text{true}}\left(m\right))}^{2}}{Q-1} \end{array}$$ (27)

$$\begin{array}{c} \text{Cov}\left(x,x_{\text{true}}\right)=\frac{\sum_{m=1}^{Q}(x_{\text{true}}\left(m\right)-{\bar{x}}_{\text{true}})\left(x\left(m\right)-\bar{x}\left(m\right)\right)}{Q-1} \end{array}$$ (28)

where *x*(*m*) denotes the voxel value of estimated low-dose image and *x*_true_(*m*) denote the voxel value of the original phantom image in the ROI, *Q* is the total number of voxels in the ROI. Hence, the UQI can be written as

$$\begin{array}{c} \text{UQI}=\frac{4\text{Cov}(x,x_{\text{true}})}{\sigma^{2}+\sigma_{\text{true}}^{2}}\frac{\bar{x}\cdot {\bar{x}}_{\text{true}}}{{\bar{x}}^{2}+{\bar{x}}_{\text{true}}^{2}}. \end{array}$$ (29)

For the UQI measure, its value ranges between 0 and 1 and UQI value more close to 1 indicates the more similarity between the reconstructed and original images.

## Results

### XCAT phantom study

#### Visualization-based evaluation

**Fig 2:**
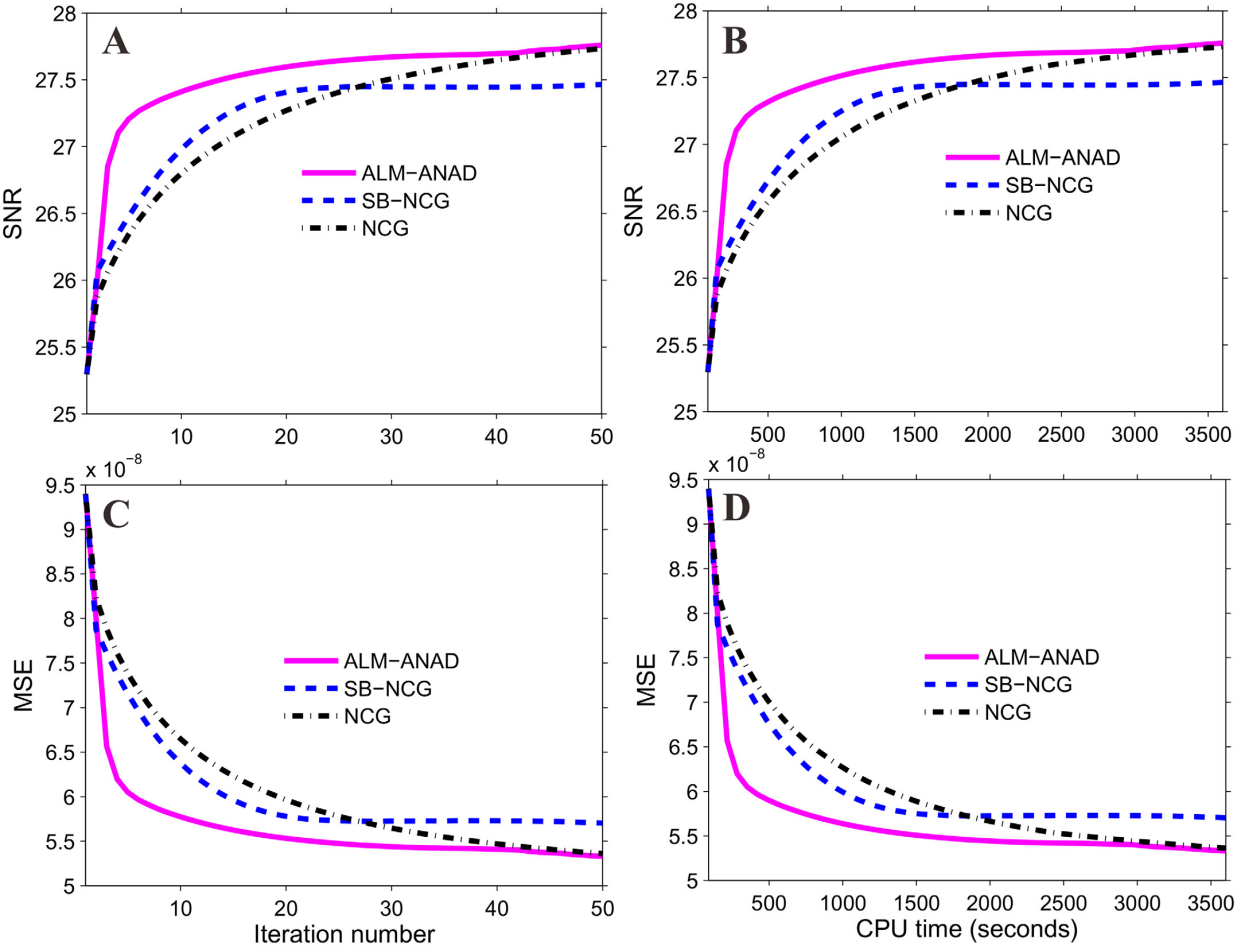
SNR and MSE measurements of three algorithms vs iteration number and CPU time, respectively. (A) is the SNR measures vs iteration number; (B) is the MSE measures vs iteration number; (C) is the SNR measures vs CPU time; and (D) is the MSE measures vs CPU time.

**Fig 3:**
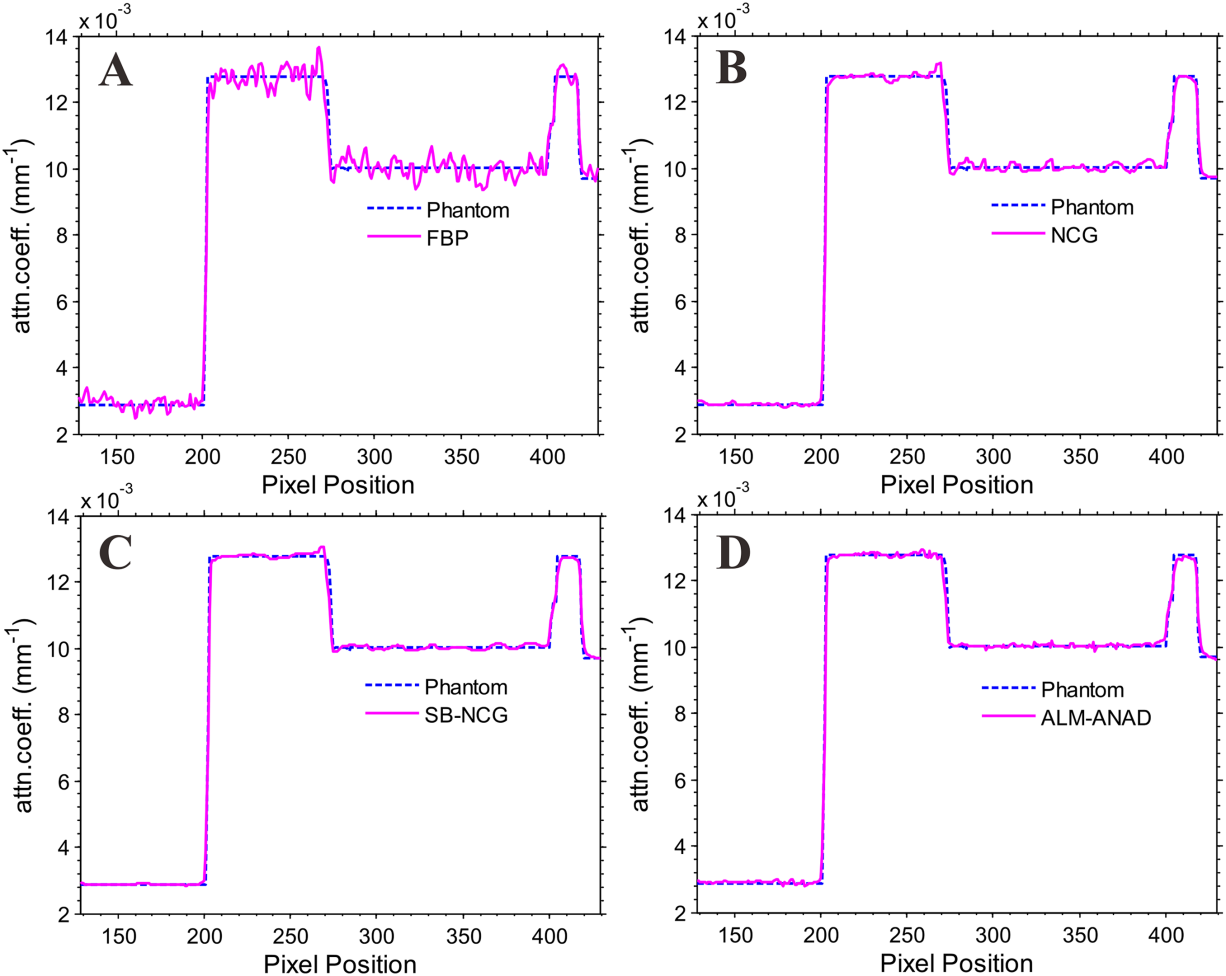
Vertical profiles (296^th^ column) of the images reconstructed from different algorithms. (A) is the result from the FBP algorithm with ramp filter; (B) is the result from the NCG algorithm; (C) is the result from the SB-NCG algorithm; and (D) is the result from ALM-ANAD algorithm. The ‘dashed line’ in each sub-figure denotes the profile from the true phantom.

Fig 1 shows the results reconstructed by four different algorithms from the same noisy sinogram data. Fig 1(A) shows the image reconstructed by the FBP method with ramp filter. Serious noise and artifacts can be observed. Fig 1(B), 1(C) and 1(D) show the results reconstructed by the NCG, SB-NCG and ALM-ANAD algorithms, respectively. We can observe that the present ALM-ANAD algorithm achieves noticeable gains over other algorithms in terms of both artifacts suppression and edge preservation. Moreover, Fig 2 illustrates the SNR measurements of three algorithms with respect to the iteration number and CPU time. It can be seen that the ALM-ANAD algorithm can yield fast converges rate than other two algorithms in terms of SNR measurements. To further visualize the difference among the three approaches, vertical profiles were drawn across the 296^th^ column, from the 296^th^ row to the 430^th^ row and are shown in Fig 3. The profile from the ALM-ANAD algorithm matches better with that from the true phantom than those from other algorithms.

#### Noise reduction measure

Table 1 shows the SNRs and MSEs of the images reconstructed by four different algorithms. The results suggest that the ALM-ANAD algorithm can achieve noticeable gains over other three algorithms in terms of the noise reduction.

**Table 1:** SNRs and MSEs of the images reconstructed by four different algorithms.

Methods	FBP	NCG	SB-NCG	ALM-ANAD
SNR(dB)	25.30	27.39	27.47	27.76
MSE(10^−8^)	9.40	5.72	5.70	5.33

#### CNR measure

For the calculation of the contrast-to-noise ratio (CNR), we selected four regions of interest (ROIs) indicated by the squares in the XCAT phantom image, which are named as the ROIA, ROIB, ROIC, and Background, respectively. Table 2 shows the CNRs of the images reconstructed by four different algorithms, respectively. It can be seen that the ALM-ANAD algorithm yields noticeable gains over other algorithms in terms of the CNR measure. Consequently, the present ALM-ANAD algorithm has the remarkable ability for identifying low-contrast regions as compared to other algorithms.

**Table 2:** CNRs of the images reconstructed by four different algorithms.

Methods	FBP	NCG	SB-NCG	ALM-ANAD
CNR (ROIA)	4.36	11.30	17.97	22.75
CNR (ROIB)	6.43	18.20	35.14	39.53
CNR (ROIC)	0.73	1.88	3.65	4.27

#### UQI measure

**Fig 4:**
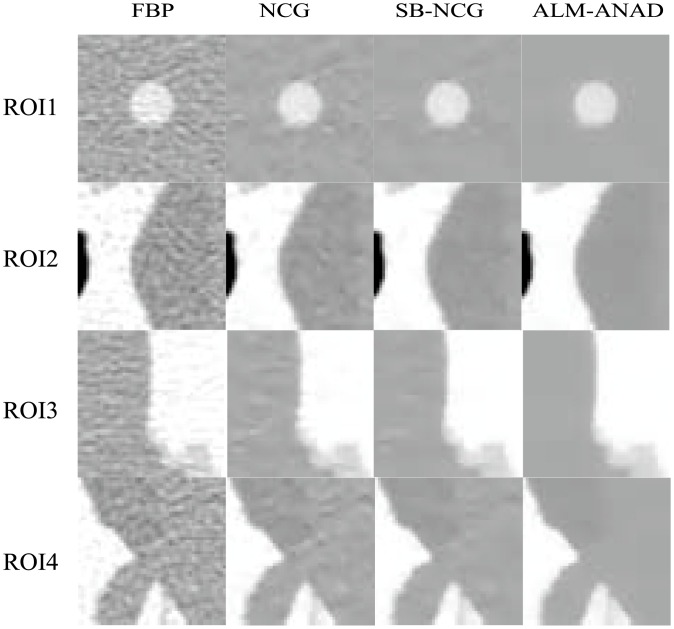
Zoomed details of the four ROIs in Fig 2.

**Fig 5:**
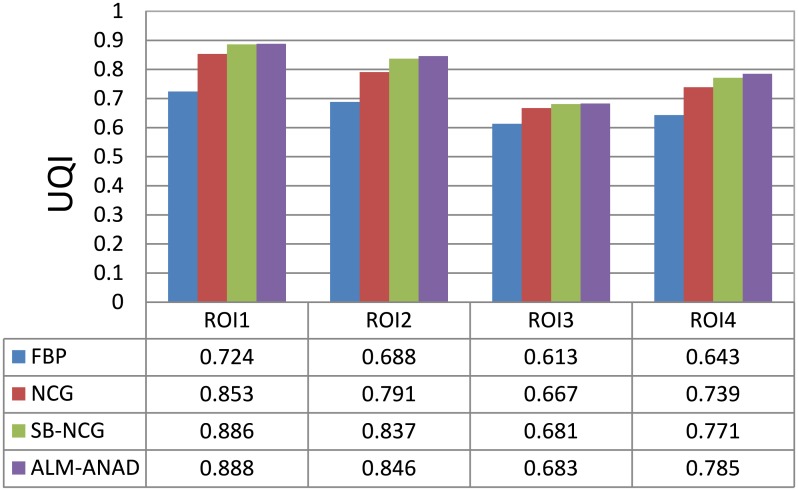
UQI measures on the four ROIs in Fig 5.

Fig 4 shows the zoomed details of four selected ROIs in Fig 1. It can be seen that the ALM-ANAD algorithm can achieve noticeable gains over other algorithms in terms of the noise-induced artifacts suppression. Furthermore, the corresponding UQI scores are illustrated in Fig 5, which shows that the gains from the ALM-ANAD algorithm are noticeable over those from the other three algorithms in terms of the UQI measure in four regions.

### Anthropomorphic torso phantom study

#### Visualization-based evaluation

**Fig 6:**
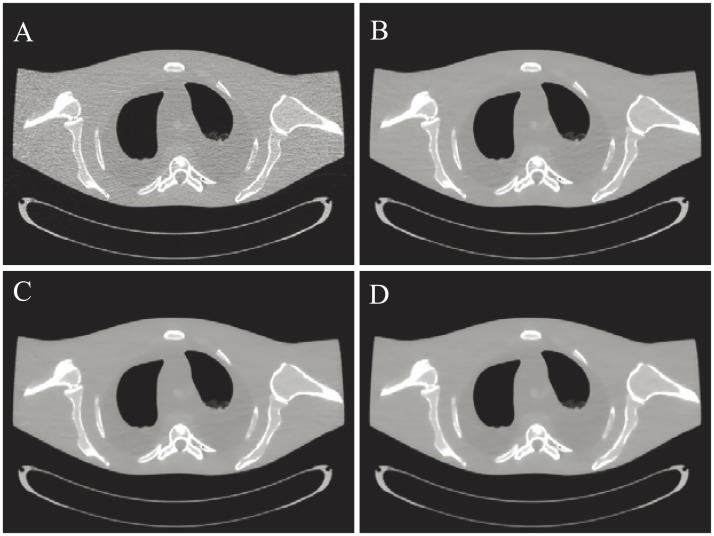
The anthropomorphic torso phantom scanned with a protocol of 40 mAs and 120 kVp. (A) is the result from the FBP algorithm with ramp filter; (B) is the result from the NCG algorithm with *β* = 1.0 × 10^5^; (C) is the result from the SB-NCG algorithm with *γ* = 2^5^, *β* = 2^9^; and (D) is the result from the ALM-ANAD algorithm with *γ* = 2^5^, *β* = 2^9^. The zoomed ROIs indicated by the rectangle are also displayed for visual appealing. All images are displayed with the same window [0.0017 0.024] mm^−1^.

**Fig 7:**
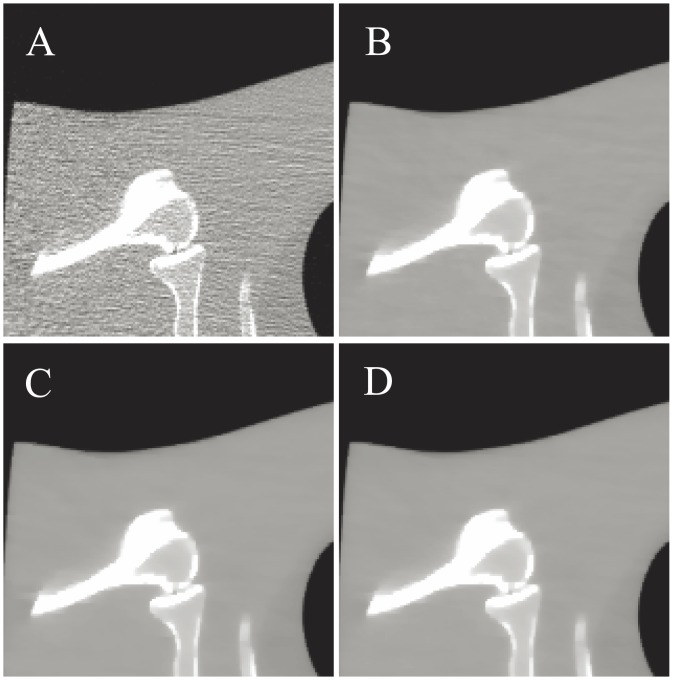
Zoomed details of the ROI in Fig 6. (A) FBP method; (B) NCG method; (C) SB-NCG method; and (D) ALM-ANAD method.

Fig 6 shows the results reconstructed by four different algorithms from the sinogram acquired with a protocol of 40 mAs and 120 kVp. Fig 6(A) shows the image reconstructed by the FBP method with ramp filter. Fig 6(B) shows the image reconstructed by the NCG algorithm. Fig 6(C) shows the image reconstructed by the SB-NCG algorithm. Fig 6(D) shows the image reconstructed by the present ALM-ANAD algorithm. To further display the gains of the ALM-ANAD method, the zoomed ROIs are shown in Fig 7. It can be observed that the ALM-ANAD algorithm achieves noticeable gains over other two algorithms in terms of successfully noise-induced artifacts suppression and edges preservation.

#### CNR measure

**Fig 8:**
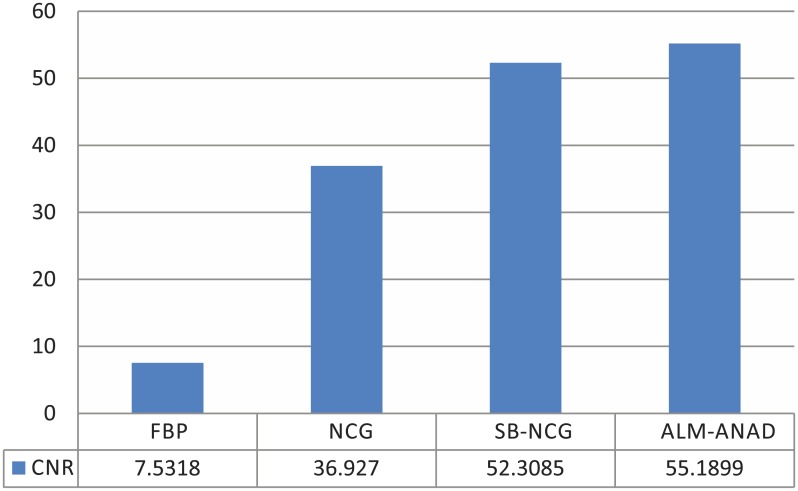
CNR measures of images reconstructed by four different methods.

To quantitative evaluation of the reconstructed images, we selected two different ROIs for the calculation of the contrast-to-noise ratio (CNR). Fig 8 shows the CNRs of the images reconstructed by four different algorithms, respectively. It can be seen that the ALM-ANAD algorithm yields noticeable gains over other algorithms in terms of the CNR measure. Consequently, the present ALM-ANAD algorithm has the remarkable ability for identifying low-contrast regions as compared to other algorithms.

#### UQI measure

**Fig 9:**
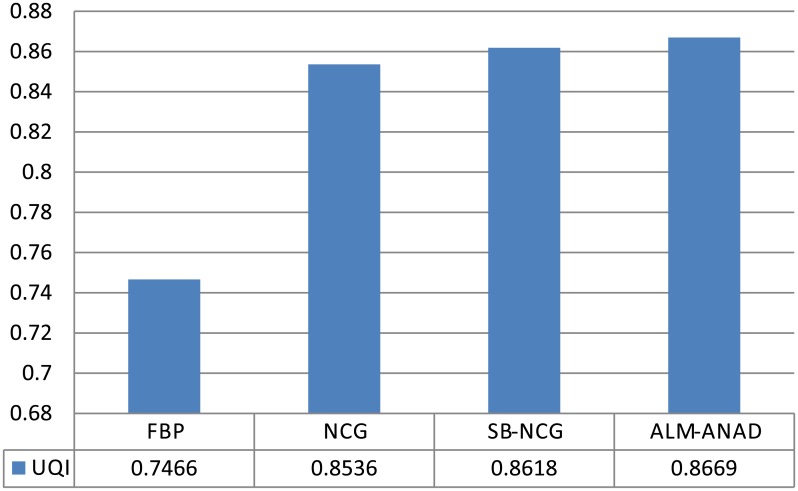
UQI measures of images reconstructed by four different methods.

Fig 7 shows the zoomed details of selected ROI the anthropomorphic torso phantom image. It can be seen that the ALM-ANAD algorithm can achieve noticeable gains over other algorithms in terms of the noise-induced artifacts suppression. Furthermore, the corresponding UQI scores are illustrated in Fig 9, which shows that the gains from the ALM-ANAD algorithm are noticeable over those from the other three algorithms in terms of the UQI measure.

## Discussion and Conclusion

The variable splitting strategy has raised increasing concerns in statistical X-ary CT reconstruction due to its appealing nature to split the regularization term and data-fidelity term [4, 12, 16, 17]. Inspired by the variable splitting strategy, in this work, we proposed an adaptive nonmonotone alternating direction optimization strategy via an efficient augmented Lagrangian multiplier approach, which was named as the ALM-ANAD algorithm. Experimental results in Section 4 have shown that the present ALM-ANAD algorithm for CT image reconstruction can achieve noticeable gains over other existing algorithms.

In general, a non-negativity constraint in CT image reconstruction is required to model the positivity of the attenuation coefficient. In this work, we did not consider this constraint in the development of the ALM-ANAD algorithm. However, mathematically we can build the following cost function with the non-negativity constraint:

$$\begin{array}{c} \min_{x}\Psi \left(Rx\right)+\frac{\beta }{2}{\left(p-Hx\right)}^{T}W^{-1}(p-Hx),s.t.\;x\geq 0. \end{array}$$ (30)

For solving Eq (30), the projected gradient method [37] can first be used by instead of the gradient descent method when updating *x* in ALM-ANAD algorithm, and after this modification all other explored techniques in the ALM-ANAD algorithm can be borrowed directly.

For the ALM-ANAD algorithm, three parameters, i.e., *s*, *β*, *γ*, were selected empirically in the present studies by visual inspection for eye-appealing results with comparison to the true phantom or the normal-dose image. In general, penalty parameter selection is a nontrivial and application-dependent task, which is usually determined by time consuming trial and error process. Meanwhile, the parameters *ρ*_min_, *δ*, *ρ*, *K* in the implementation of the ANAD algorithm were adaptively determined using the nonmonotone line search technique described in [20, 21]. In practice, more theoretical insight in optimizing the parameters is necessary for the ALM-ANAD algorithm, which is an interesting topic for future research.

All the PWLS-based algorithms in the studies were implemented in Matlab 7.9 (The Math Works, Inc.) programming environment. The codes were run on a typical desktop computer with Intel Xeon X5647 Processor, 2.93 GHz and 24 GB of RAM memory. To reconstruct an image with size of 512 × 512, the ALM-ANAD algorithm took approximately 0.4 min per iteration while the SB-NCG and NCG algorithm took approximately 0.5 and 0.7 min, respectively. The gain from the ALM-ANAD algorithm is noticeable over that from the NCG algorithm. However, it is worth to notice that because the splitting-based algorithms suffer from the cost of manipulating and storing auxiliary constraint variable [4, 11, 12, 16, 17], the ALM-ANAD algorithm for CT image reconstruction has the drawback of heavy memory load. Practically, the present ALM-ANAD algorithm needs additional memory requirements as compared with the classical NCG algorithm, especially in the case of 3D image reconstruction. In further research, more advanced accelerate methods based on the ALM-ANAD algorithm should be explored, such as ordered subset and GPU based speed-up techniques, which is an interesting topic. Another draw back of the present ALM-ANAD algorithm is that it may lead to over-smooth around edges or boundaries as described in Fig 3(D). To preserve edges in the reconstructed images, effective iterative reconstruction method with reasonable parameter selection is necessary that enables one to achieve a clinically acceptable image with as low as possible mAs without compromising quality, which is an interesting topic for future research.

In this work, the present ALM-ANAD algorithm was only focusing on low-dose CT image reconstruction. Meanwhile, the present algorithm can also be used in other applications, including positron emission tomography (PET) [38], single photon emission CT [39], mobile landmark search framework [40], codebook compression [41], mobile visual location recognition [42, 43]. This is an interesting topic for future research.

## Supporting Information

S1 Text Appendix 1: Adaptive Nonmonotone Line Search. (PDF)

S2 Text Appendix 2: Convergence Analysis of ALM-ANAD Algorithm. (PDF)
