# Supplementary material for: An Efficient Augmented Lagrangian Method for Statistical X-Ray CT Image Reconstruction
Source: PLoS One. 2015 Oct 23;10(10):e0140579. doi: 10.1371/journal.pone.0140579 (PMC4619856; doi:10.1371/journal.pone.0140579)
Supplement: S1 Text — (PDF) [file pone.0140579.s001.pdf]

Appendix 1: Adaptive Nonmonotone Line Search

The adaptive nonmonotone line search for smooth function was proposed by Dai and Fletcher in [20]. In this method, as described in detail previously [21], ψ_r is a reference function value, and it must be improved at each iteration such that

$$\psi(x_k + \alpha d_k) \leq \psi_r + \alpha\delta\nabla\psi(x_k)^T d_k \tag{1}$$

where d_k is a descent direction, and $\delta \in (0, 1)$ is a given constant and $\alpha > 0$ is the tried stepsize. As described in detail previously [28], the sequence of α should decrease in this line search procedure, starting with $\alpha = 1$, until the test condition (1) is satisfied. As described in detail previously [28], ψ_{best} denotes the current least value of the objective function over all past iterates, that is, $\psi_{best} = \min_{1\leq i\leq k} \psi(x_i)$ at the k-th iteration. As described in detail previously [28], l denotes the number of iterations since the value of ψ_{best} was obtained. As described in detail previously [28], the candidate function ψ_c denotes the maximum value of the objective function since the value of ψ_{best} was found.

The updating strategy for the reference function value f_r is described in detail previously [28]. Suppose that K is a preset positive integer. To allow $\psi(x_k) \geq \psi(x_0)$ on early iterations, we can set $\psi_r = +\infty$ initially as described in detail previously [28]. If there is a better function value in K iterations, then the value of ψ_r remains unchanged. Otherwise, if $l = K$, we reset the reference function value ψ_r to ψ_c and reset ψ_c to the current value $\psi(x_k)$ as described in detail previously [28]. A more precise statement is presented in the ANAD algorithm.
