# Supplementary material for: An Efficient Augmented Lagrangian Method for Statistical X-Ray CT Image Reconstruction
Source: PLoS One. 2015 Oct 23;10(10):e0140579. doi: 10.1371/journal.pone.0140579 (PMC4619856; doi:10.1371/journal.pone.0140579)
Supplement: S2 Text — (PDF) [file pone.0140579.s002.pdf]

Appendix 2: Convergence Analysis of ALM-ANAD Algorithm

The convergence of ALM has been thoroughly studied [22,23], so the convergence of the present ALM-ANAD algorithm relies on the convergence of ANAD algorithm. By extending Dai and Fletcher's proof [20], we establish the convergence results of ANAD algorithm.

Firstly, we impose the following assumptions on the function $\phi(x,y)$.
Assumption1 There exists $L>0$, such that at any given y, and for all x and $\tilde{x}$,

$$\|\nabla_1\phi(x,y)-\nabla_1\phi(\tilde{x},y)\|_2 \le L\|x-\tilde{x}\|_2. \tag{1}$$

Assumption 2 The function $\phi(x,y)$ is bounded below, i.e., there exists $C>0$ such that for all (x,y),

$$\phi(x,y)\ge -C. \tag{2}$$

The convergence result of ANAD algorithm relies on the following lemma. For notational simplicity, let us define $\phi_k(\cdot)=\phi(\cdot,y_k)$ and $\nabla\phi_k(\cdot)=\nabla_1\phi(\cdot,y_k)$.
Lemma 1 Let α_k satisfies condition (**??**) in ANAD algorithm, for all $k>0$, we have

$$\alpha_k \ge \min\left\{1, \frac{2(1-\delta)\theta_1}{L}\frac{|\nabla\phi_k(x_k)^T d_k|}{\|d_k\|^2}\right\} \tag{3}$$

Proof. In fact, if $\alpha=1$ satisfies condition (**??**), then we have $\alpha_k=1$. Otherwise, there exists $\rho\in[\theta_1,\theta_2]$ for which $\frac{\alpha_k}{\rho}>0$ fails to satisfy condition (**??**), it follows that

$$\phi_k(x_k+\frac{\alpha_k}{\rho}d_k) > \phi_r + \delta\frac{\alpha_k}{\rho}\nabla\phi_k(x_k)^T d_k. \tag{4}$$

On the other hand, by the mean-value theorem and Lipschitz condition, we have

$$\begin{aligned}\phi_k(x_k+\tfrac{\alpha_k}{\rho}d_k)-\phi_k(x_k) &= \textstyle\int_0^{\frac{\alpha_k}{\rho}}\langle\nabla\phi_k(x_k+td_k)-\nabla\phi_k(x_k), d_k\rangle dt + \tfrac{\alpha_k}{\rho}\nabla\phi_k(x_k)^T d_k \\ &\le \tfrac{L}{2}\left(\tfrac{\alpha_k}{\rho}\right)^2\|d_k\|^2 + \tfrac{\alpha_k}{\rho}\nabla\phi_k(x_k)^T d_k.\end{aligned} \tag{5}$$

Combing the above two inequalities, we can find that (3) holds.

Then, the convergence theorem result of the ANAD algorithm can be described as follows.
Theorem 1 Let $\{(x_k,y_k)\}$ be a sequence generated by the ANAD algorithm. Then any accumulation point of the sequence $\{(x_k,y_k)\}$ is a stationary point, that is,

$$\begin{cases}\liminf\limits_{k\to\infty}\|\nabla_1\phi(x_k,y_k)\|_2=0,\\ \nabla_2\phi(x_k,y_k)\ni 0.\end{cases} \tag{6}$$

Proof. We need to establish the two relationships given in equation (6). As the definition $y_k=\arg\min\limits_{y}\phi(x_k,y)$ in the ANAD algorithm, $0\in\nabla_2\phi(x_k,y_k)$ always holds. Thus, it

suffices to show that the second equation holds in (6). By contradiction, for all $k > 0$, we have $\langle \nabla \phi_k(x_k), d_k \rangle \leq -\epsilon$ for some $\epsilon > 0$. In this case, we can conclude that there exists an infinite subsequence I such that for $k_i \in I$, the values of ϕ_r on iterations k_i are strictly monotonically decreasing. Let $\phi_r^{k_i}$ denote the value ϕ_r on iteration k_i, we have

$$\phi_{k_i}(x_{k_i+1}) \leq \phi_r^{k_i} + \delta\alpha_{k_i} \langle \nabla \phi_{k_i}(x_{k_i}), d_{k_i} \rangle \leq \phi_r^{k_0} + \delta \sum_{j=k_0, j\in I}^{k_i} \alpha_j \langle \nabla \phi_j(x_j), d_j \rangle \tag{7}$$

Furthermore, from the definition of d_k and Lemma 1, we have $\langle \nabla \phi_k(x_k), d_k \rangle \leq -\frac{1}{\rho_{\max}} \|d_k\|^2$ and $\alpha_k \geq \min\left\{1, \frac{2(1-\delta)\theta_1}{L\rho_{\max}}\right\}$. So, we can derive that

$$\phi_r^{k_0} - \phi_{k_i}(x_{k_i}) \geq \delta \sum_{j=k_0, j\in I}^{k_i} \alpha_j \langle \nabla \phi_j(x_j), d_j \rangle \geq \epsilon\delta \sum_{j=k_0, j\in I}^{k_i} \min\left\{1, \frac{2(1-\delta)\theta_1}{L\rho_{\max}}\right\} \tag{8}$$

Since $\phi(x, y)$ is bounded below, let $i \to \infty$, we get $\infty > \phi_r^{k_0} - \phi_{k_i}(x_{k_i}) \to \infty$. This is a contradiction. Hence, $\lim_{k\to\infty} \inf \|\nabla_1 \phi(x_k, y_k)\|_2 = 0$, which completes the proof.

Finally, as indicated before, the convergence of ALM-ANAD algorithm follows from that of ANAD algorithm.
